# The value of intraoperative indocyanine green angiography in microvascular decompression for hemifacial spasm to avoid brainstem ischemia

**DOI:** 10.1007/s00701-022-05389-2

**Published:** 2022-10-27

**Authors:** Ahmed Al Menabbawy, Ehab El Refaee, Loay Shoubash, Marc Matthes, Henry W. S. Schroeder

**Affiliations:** 1 Department of Neurosurgery, University Medicine Greifswald, Greifswald, Germany; 2 Department of Neurosurgery, Cairo University, Giza, Egypt

**Keywords:** Microvascular decompression, Hemifacial spasm, Brainstem ischemia, Indocyanine green videoangiography, Sling decompression technique, Brainstem perforators

## Abstract

**Purpose:**

Despite being rarely reported, ischemic insults resulting from compromising small brainstem perforators following microvascular decompression (MVD) remain a potential devastating complication. To avoid this complication, we have been using indocyanine green (ICG) angiography intraoperatively to check the flow within the small brainstem perforators. We aim to evaluate the safety and usefulness of ICG videoangiography in MVD.

**Methods:**

We extracted retrospective data of patients who received ICG videoangiography from our prospectively maintained database for microvascular decompression. We noted relevant data including demographics, offending vessels, operative technique, outcome, and complications.

**Results:**

Out of the 438 patients, 15 patients with a mean age (SD) of 53 ± 10.5 years underwent intraoperative ICG angiography. Male:female was 1:1.14. The mean disease duration prior to surgery was 7.7 ± 5.3 years. The mean follow-up (SD) was 50.7 ± 42.0 months. In 14 patients, the offending vessel was an artery, and in one patient, a vein. Intraoperative readjustment of the Teflon pledget or sling was required in 20% (3/15) of the cases. No patient had any sort of brainstem ischemia. Eighty percent of the patients (12/15) experienced complete resolution of the spasms. 86.7% (13/15) of the patients reported a satisfactory outcome with marked improvement of the spasms. Three patients experienced slight hearing affection after surgery, which improved in two patients later. There was no facial or lower cranial nerve affection.

**Conclusion:**

Intraoperative ICG is a safe tool for evaluating the flow within the brain stem perforators and avoiding brainstem ischemia in MVD for hemifacial spasm.

**Supplementary Information:**

The online version contains supplementary material available at 10.1007/s00701-022-05389-2.

## Introduction

Microvascular decompression (MVD) is the most effective modality for the treatment of hemifacial spasm (HFS) with a low rate of complications [16, 18, 24]. However, compressions caused by large or even megadolichoectatic vertebral arteries are challenging pathologies that sometimes may not sufficiently be decompressed by simply interposing Teflon pledgets [6, 8][8, 12]. For this reason, in some patients, transposition of the compressing vessel is inevitable to strive for adequate nerve decompression [6, 8, 13]. During the transposition of the compressing vessel, small brainstem perforators might be endangered. It is notable that even the slightest stretching or kinking might impede adequate blood flow and subsequent perfusion of vital areas supplied by these tiny vessels. In some cases, and in spite of the intraoperative visual impeccability of the perforators, blood flow and tissue perfusion impairment might still take place. Intraoperative Doppler might be helpful in evaluating larger arteries, but blood flow in small perforators is very difficult to be assessed. Such challenges might result in ischemic insults to the brainstem and drastic sequelae [10, 19].

After having experienced one patient with a brainstem ischemia after transposing the vertebral artery with a Gortex sling, we adopted the intraoperative indocyanine green (ICG) angiography [17] whenever a brainstem perforator is at risk of hypoperfusion. Since ICG in MVD has not been adequately reviewed in the literature, we present our technique assessment and experience with ICG in our series and evaluate its influence on the outcome and complication rates [23].

## Methods

### Study design and data collection

This study was approved by the local institutional ethical committee and was performed in accordance with the 1964 Helsinki Declaration and its later amendments. Written patients’ consents were obtained preoperatively. All data were collected prospectively and recorded on our institutional database. The retrospective review of our prospectively maintained database took place for patients undergoing MVD for HFS. Out of 438 patients, only patients who received intraoperative ICG angiography in the period between April 2005 and May 2021 were analyzed. The following data were extracted: age at the time of operation, sex of the patient, duration of the disease, the side of HFS, the offending vessels, the technique for decompression (shredded Teflon or transposing with a sling), complications, and outcome. All these data are available in Table 1. We also reviewed the operative reports concerning whether a correction/repositioning of the decompression (either position of the Teflon pledgets or sling) was performed following intraoperative ICG angiography or not.

**Table 1:** Showing patients’ demographics, surgical data, and postoperative outcome

Patient Nr	Sex	Age	Duration of symptoms	Site	Compressing vessel	Sling/Teflon	VB Dm (mm)	L	TV	ReC	Post Op improvement	Post Op Comp	Brainstem ischemia	Hearing affection	Facial palsy	FU (m)
1	F	48	1	Left	AICA	Teflon	2.3	No	Yes	No	Fair	Yes	No	Yes	No	111
2	F	58	8	Left	Vein	Sling	3.9	No	No	No	Excellent	No	No	No	No	96
3	M	65	17	Left	Vertebral + AICA	Teflon	3	Yes	Yes	No	Excellent	No	No	Yes	No	97
4	F	30	4	Right	Vertebral + PICA	Teflon	NA	NA	NA	No	Excellent	No	No	No	No	71
5	F	70	4	Right	PICA	Teflon	5	No	No	No	Excellent	No	No	No	No	94
6	M	46	11	Right	AICA	Sling	3	Yes	No	No	Excellent	No	No	No	No	101
7	M	58	11	Right	AICA + PICA	Teflon	2	Yes	No	No	Excellent	No	No	No	No	75
8	F	58	15	Left	PICA + Arachnoid bands	Teflon	3	No	No	Yes	Fair	No	No	No	No	30
9	M	54	14	Right	PICA	Teflon	4	Yes	Yes	No	Excellent	No	No	No	No	28
10	M	63	8	Left	Vertebral	Sling	7.5	Yes	Yes	Yes	Excellent	No	No	No	No	20
11	M	46	5	Left	AICA	Teflon + coagulation of ectatic vein	2	No	No	No	Excellent	No	No	No	No	19
12	F	59	2	Right	Vertebral + AICA	Sling	5	Yes	Yes	Yes	Excellent	No	No	No	No	10
13	F	55	12	Right	AICA	Sling	2.9	No	No	No	Excellent	Yes	No	Yes	No	4
14	M	45	1	Left	Vertebral + PICA	Sling	5.3	Yes	Yes	No	Good	No	No	No	No	2
15	F	40	3	Right	PICA	Sling	2.9	No	No	No	Excellent	No	No	No	No	2

*AICA*, anterior inferior cerebellar artery;* Comp.*, complications; *F*, female;* FU (m)*, follow-up in months; *L*, laterality of the vertebral artery; *M*, male; *NA*, not available; *PICA*, posterior inferior cerebellar artery; *Post Op*, postoperative; *ReC*, recorrection of the sling or shredded Teflon; *TV*, tortuous vessel, *VB*,* Dm* maximum diameter of the vertebrobasilar arteries; Post-operative improvement, Excellent, 100% resolution of the spasms; Good, > 90% improvement of the spasms; Fair, 50–90% improvement of the spasms; and Poor, < 50% improvement of the spasms

Additionally, measurements of the vertebrobasilar arterial system were done on MRI images. We measured the maximum diameter of the vertebrobasilar arterial system on the side of the lesion. We also noted the presence of tortuosity of the vertebrobasilar system on the site of the lesion and accordingly identified and reported criteria for dolichoectasia of the vertebrobasilar arterial system whenever present according to the Smoker’s criteria. The criteria included a diameter of 4.5 mm or more of the vessel (ectasia), evident tortuosity, or laterality of the basilar artery on the site of the disease (Dolicho) [3, 20].

### Outcome measurements

The outcome was divided into 4 grades: (1) excellent (hemifacial spasm completely absent), (2) good (hemifacial spasm > 90% resolved), (3) fair (hemifacial spasm 50–90% resolved), and (4) poor (hemifacial spasm < 50% resolved). Ninety percent improvement means that no visible spasms occurred, or rarely slight spasms were observed that did not occur daily. Complications in general were reported and especially facial weakness or hearing loss following the MVD.

### Surgical technique and strategy

All MVDs were performed by the senior author of this study through a lower retrosigmoid approach in a supine position under the monitoring of facial electromyography (EMG) and of brainstem auditory evoked potentials (BAEP). The lateral spread response was recorded routinely. Depending on the anatomical situation, the decompression was achieved by transposing the vessel using shredded Teflon. When the offending vessel was causing a major compression which could not be relieved by shredded Teflon pledgets alone, a Gortex or Teflon sling was used to transpose the vessel. The sling was fixed to the basal dura with the aid of an aneurysm clip or suture. Whenever a compromise of blood flow within the offending vessel or a nearby perforator was suspected, we performed an intraoperative ICG videoangiography and accordingly the needed corrections of the decompression.

## Results

Out of the total 438 patients’ data entries, intraoperative ICG videoangiography was performed in 15 patients. A summary of patient demographics, offending vessels, complications, follow-up, and outcome is shown in Table 1. The comparison of the ICG angiography group vs the non-ICG angiography group is tabulated in Table 2.

**Table 2:** Angiography group vs non-angiography group

	Angiography group	Non-angiography group	Total
Number	15	423	438
Mean age (SD)	53 ± 10.5	55.2 ± 11.4	55.1 ± 11.6
Males (%)	7 (46.67%)	162 (38.3%)	169 (38.6%)
Mean symptoms duration (SD)	7.73 (5.34)	7.9 (5.2)	7.89 (5.2)
Left sided	7 (46.67%)	259 (61.2%)	266 (60.7%)
Dolicoectasia	8 (60%)	21 (4.96%)	29 (6.62%)
Vessel transposition (sling technique)	7 (46.67%)	37 (8.74%)	44 (10.0%)
Excellent outcome (cure)	12 (80%)	308 (72.8%)	320 (73.1%)
Facial palsy	0	35 (8.27%)	35 (7.99%)
Hearing affection	3 (20%)	55 (13.0%)	58 (13.2%)
Brainstem ischemia	0	1 (0.24%)	1 (0.23%)

The ages of the patients ranged from 30 to 70 years with an average of 53 ± 10.5 (SD) years. There were eight females (53.3%) and seven males (46.7%). Duration of the disease until the date of surgery ranged from 1 to 17 years with a mean duration of 7.7 ± 5.3 years. The spasm was on the right side in eight patients (53.3%).

The anterior inferior cerebellar artery (AICA) was the offending vessel in four patients (26.7%), the posterior inferior cerebellar artery (PICA) in three patients (20%), the vertebral artery (VA) in one patient (6.7%), VA + AICA and VA + PICA in two patients each (13.3%), AICA + PICA, AICA + arachnoid, and pure venous compression in one patient each (6.7%) [7].

Nine patients (60%) fulfilled one or more of the criteria of dolichoectasia of the vertebrobasilar system. Analysis of the diameter of the vertebrobasilar system on MR images revealed that four patients had a maximum measured diameter > 4.5 mm. In eight patients, radiological tortuosity of the vertebrobasilar system was evident and evident lateralization of the vertebrobasilar system was noted in seven of these 8 patients with evident tortuosity as shown in Table 1 [20, 21].

Decompression by transposition of the offending vessel with sling technique was necessary in seven patients (46.7%). In another seven patients (46.7%) shredded Teflon was used, while in 1 patient, shredded Teflon together with coagulation of an ectatic vein were performed (patient number 11, Table 1). The sling technique was also used in the patient with venous compression where the vein could be successfully displaced using a Teflon sling (patient number 2, Table 1). Readjusting the decompression following ICG angiography was necessary in 3 patients (20%) due to evident delayed filling of one or more perforators. In 1 patient, the shredded Teflon had to be repositioned, and in the other 2 patients, re-positioning of the sling was necessary where also dolichoectasia was evident (Table 1).

The follow-up period ranged from 2 to 111 months with an average of 50.7 ± 42.0 (SD) months. No signs or symptoms or even radiological evidence of brainstem ischemia was noted in any patient. In the two patients in whom readjustment of the sling was performed, complete resolution of the spasms was evident at the last available follow-up as shown in Table 1. In the patient where re-positioning of the shredded Teflon was done, she experienced fair improvement of the symptoms after her second operation. In total, 12 patients (80%) had excellent outcomes with 100% resolution of the symptoms at the last follow-up. One patient (6.7%) had a good outcome with > 90% resolution of the spasms. This makes the favorable outcome reach 86.7%. Two patients experienced only some improvement of the symptoms with a fair outcome (13.3%), and no patients had a poor outcome with less than 50% improvement of the spasms.

In the three patients, where re-correction of the sling or shredded Teflon was done, no complications occurred. Among the whole series, only 3 patients suffered from a postoperative complication which was a slight ipsilateral hearing affection on the side of the operation. In 2 patients, this hearing loss was temporary, and only in 1 patient (patient 3), the hearing loss was permanent but with remaining useful hearing. No postoperative facial nerve weakness was observed in any of the patients.

## Case presentation

**Fig. 1:**
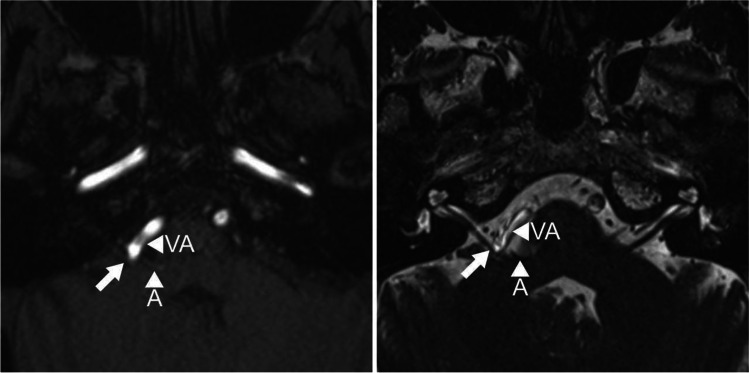
Preoperative MR imaging with TOF (left) and CISS (right) axial images showing a compression of the facial nerve root exit zone (arrow) by AICA (A) and VA (VA)

We present one of the encountered patients (patient 9) where correction of the applied sling was mandatory to keep an adequate blood flow through a small brainstem perforator. The patient is a 59-year-old female who had suffered from a progressive right hemifacial spasm for 2 years. She tried Botox therapy but was unsatisfied with its outcome. MR images showed clearly a compression of the facial exit zone by AICA and VA loop (Fig. 1).

**Fig. 2:**
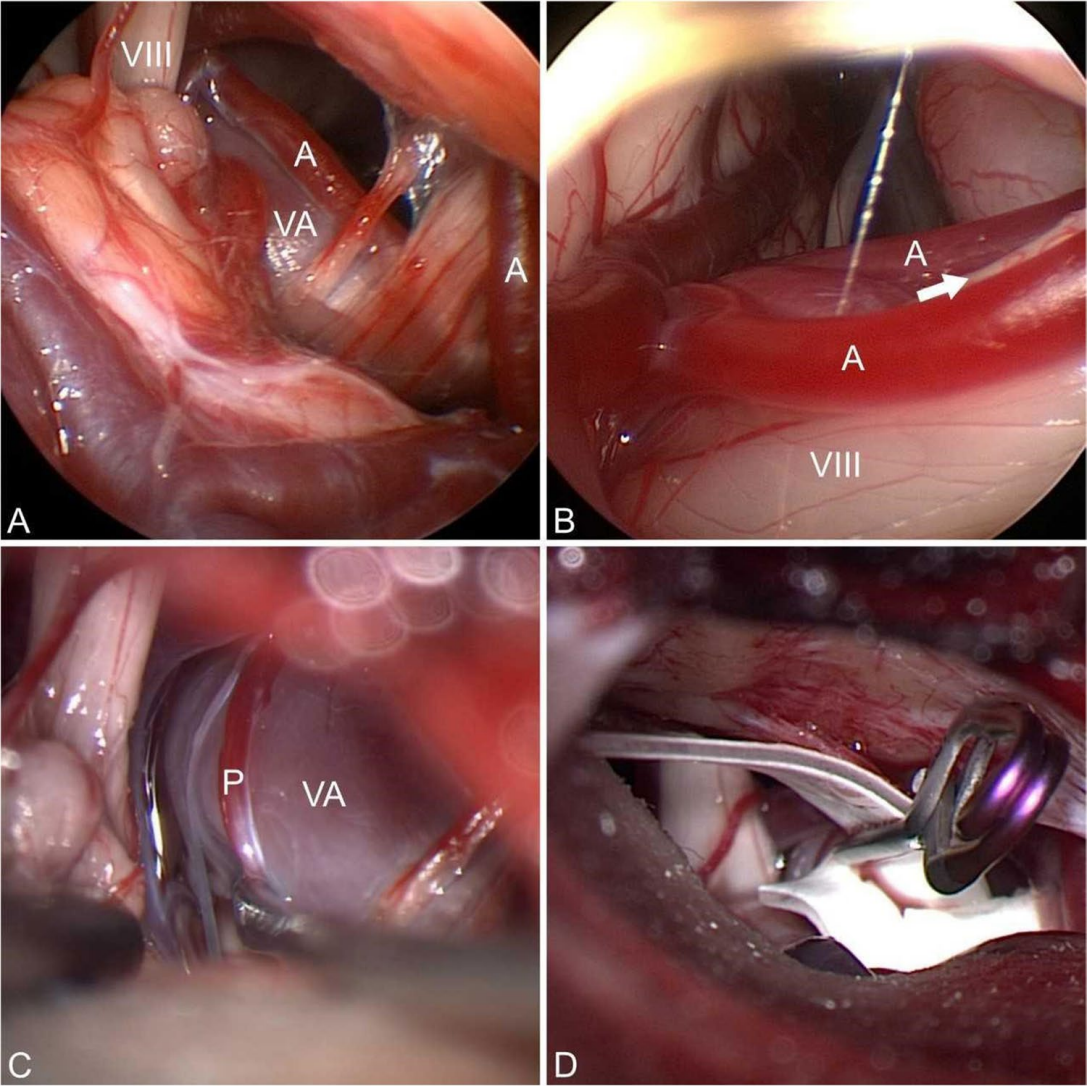
Intraoperative images. **A** Severe compression of the facial nerve by a dilated VA (VA), VIII (vestibulocochlear nerve), and A (AICA). **B** Endoscopic inspection after the elevation of the VA shows an additional compression of the facial REZ by AICA (A) and VIII (vestibulocochlear nerve). **C** Tiny perforator (P) running over VA (VA). **D** Decompression of the VA by sling which was fixed to the dura with a Yasargil mini clip

**Fig. 3:**
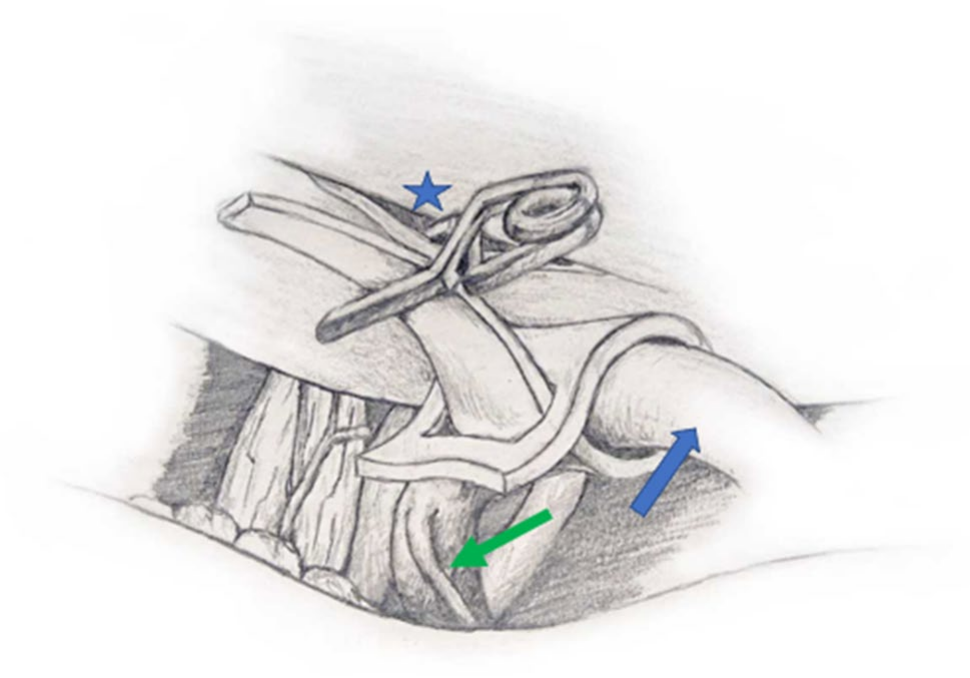
Sketch illustrating the sling decompression/vessel transposition technique of the vertebral artery (blue arrow) using a microvascular clip attached to a pocket of the dura of the skull base (blue star). The endangered perforating artery is referred to with a green arrow

**Fig. 4:**
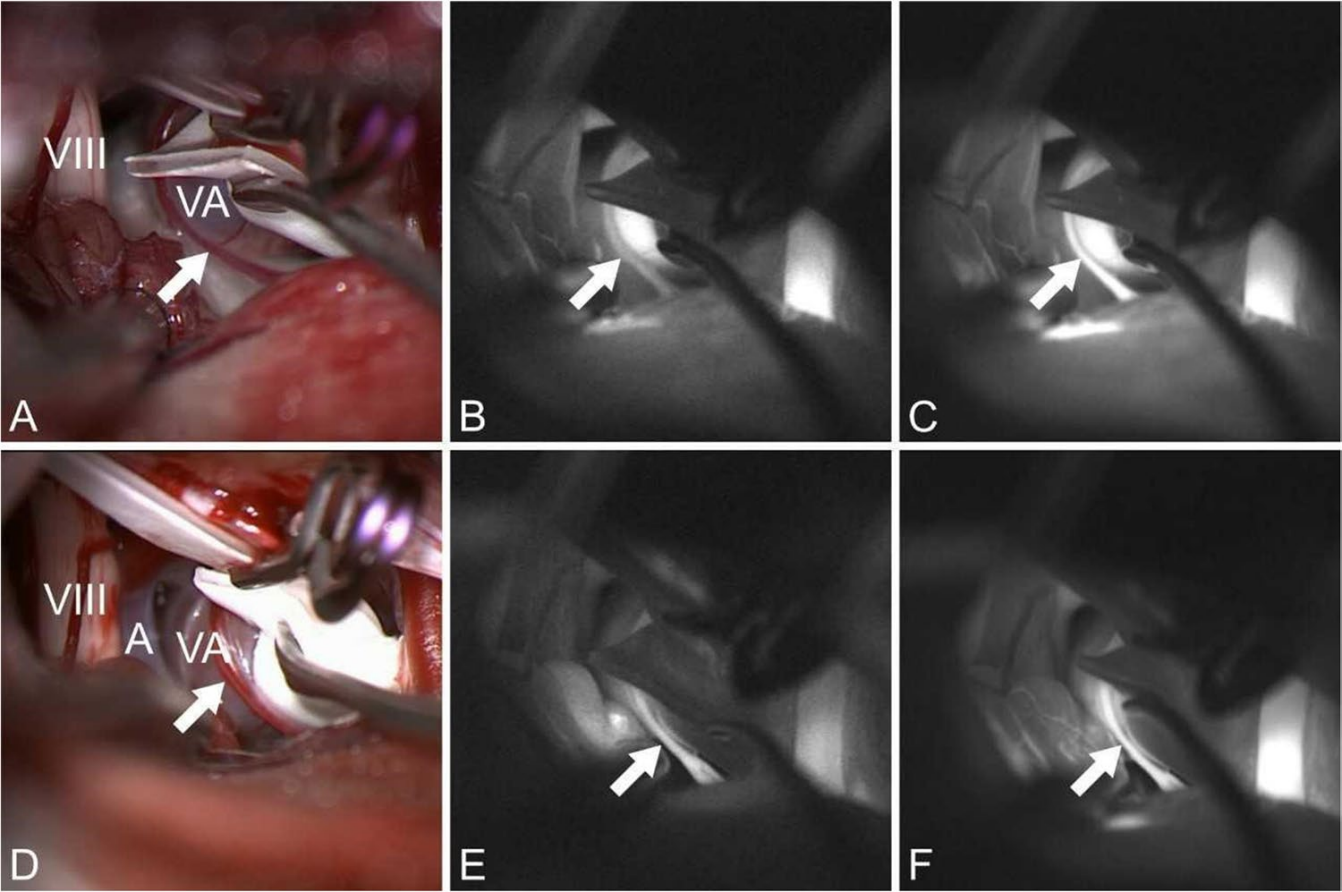
Microscopic and intraoperative indocyanine green angiography images over time. **A** The small perforating artery (arrow) looked somewhat stretched after the transposition of the VA (VA) by the Gortex sling (VIII, vestibulocochlear nerve). **B** and **C** Preadjustment time sequence of ICG angiography images showing a delayed filling of the perforator (arrow) compared to VA. Initially, the perforator is still dark although VA is already bright. Only in the late image, the perforator and VA and other vessels are bright. **D** reduced stretch on the perforator (arrow) after the release of the sling. **E** and **F** Postadjustemt time sequence images of ICG angiography showed a timely filling of the perforator (arrow) compared to VA

Intraoperatively, a severe compression of the facial nerve by a prominent vertebral artery was seen. Endoscopic inspection under the slight elevation of the vertebral artery loop using a microdissector could identify additionally a clear compression of the facial nerve at the brainstem by AICA (Fig. 2B). The vertebral artery (VA) itself pushed the AICA toward the facial nerve and compressed it (double and sandwich compression). An attempt to decompress the VA by placing shredded Teflon failed. Therefore, a Gortex sling was created to pull the vessel laterally and posteriorly away from the facial root exit zone (REZ). Dissection of the VA in the area where the sling was placed revealed a tiny perforator (Fig. 2C). The sling was placed around the VA and pulled toward the skull base (Fig. 3). After the preparation of a dura pocket between the internal auditory canal and the jugular foramen, the sling was fixed to the dura with a Yasargil mini clip (Figs. 2D and 3). The vertebral artery could be drawn very nicely to the skull base so that there was no contact with the facial/vestibulocochlear nerve complex. However, the small perforating artery looked somewhat stretched (Fig. 4A). Therefore, the decision was made to check the blood flow with the aid of ICG angiography. The angiography showed a delayed filling compared to AICA and VA (Fig. 4B and C and Video 1). Therefore, the clip was opened, and the sling was a little bit released so that the stretch on the perforator was reduced. After the readjustment of the sling, the ICA angiography showed a timely filling at the same time as AICA and VA (Fig. 4E and F). Then, the AICA loop was mobilized and decompressed with shredded Teflon. The postoperative course was uneventful with no complications. The patient experienced a complete resolution of the symptoms immediately after the operation. At the 10-month follow-up, the patient was spasm-free with normal hearing and facial function.

## Discussion

The main finding of this study is stressing on the safety and usefulness of intraoperative ICG angiography in MVD especially in cases with complicated anatomy of the posterior fossa and dolichoectasia of the vertebrobasilar system. The performance of ICG angiography in these cases can adequately assess the blood flow in the small perforators and guide further adjustments in the MVD technique accordingly (Fig. 4 and Video 1).

Intraoperative live-time ICG angiography during MVD is not adequately reported in the literature. Moreover, its usefulness in guiding the surgeon to readjust the decompression technique has not been adequately evaluated. Despite the effectiveness of MVD in treating cranial nerve–vascular compression pathologies, some cases remain challenging, and decompression using shredded Teflon or Teflon pledgets is sometimes inadequate in achieving appropriate decompression. In fact, anatomical variations of the posterior fossa anatomy and the presence of abnormal dilatation and tortuosity of the posterior circulation may hinder or even complicate the procedure of neurovascular decompression. In one study, the authors analyzed the different conflicting vessels responsible for the HFS in the literature as well as their own cases. Some studies among this review stressed on the role of posterior fossa conformation in causing HFS. Also, the conflicting vessel or vessels in cases of double compression pathology might add to the complexity of the operation. Although most of the compressions are caused by arterial conflicting vessels (usually PICA or AICA), we had only one case of pure venous compression and another case with venous compression combined with vertebral artery compression, and some series reported up to 4.5% of cases with venous compression as the conflicting vessel [2, 4–6, 11, 14, 16].

At this point, more aggressive transposition techniques using larger Teflon pledgets or displacing the compressing artery using a sling technique are required to achieve an adequate decompression. We prefer the sling technique where the sling is fixed with an aneurysmal clip to the dura of the skull base, and easy adjustment of the clip can be done [1, 6, 22]. However, great care must be given to small perforating branches that might be compromised by the relocation of the compressing mother vessel. That is why some authors questioned the safety of performing such sling decompressions like in a recent study because of the possibility of brainstem ischemia [10, 19].

In transposition maneuvers and despite extreme care, some stretch might be exerted on one or more of the brainstem perforators especially in cases of dolichoectatic basilar and vertebral arteries. In such cases, we recommend intraoperative ICG videoangiography in order to evaluate the blood flow in the small perforators, which the surgeon’s eye and Doppler sonography cannot assess.

Although the decision for performing the ICG angiography was taken by the senior author whenever he suspected a compromise of the perforator(s), it is important to note that in these 15 patients, and after retrospectively analyzing them, the incidence of patients fulfilling one or more of the criteria of vertebrobasilar dolichoectasia was quite high reaching 60% (8/15 patients). This is almost 10 times the normal incidence reported in other studies and in our own series (Table 2) where the incidence of the dolichoectatic vertebrobasilar system ranged from 0.5 to 6.6% [6, 8, 9] [11]. Additionally, in seven of the 15 patients, the sling technique was needed to achieve adequate decompression. We usually reserve this technique only for difficult and complex compressions, which means that the cases in this series were technically challenging.

On the other hand, the outcome of the patients is comparable to that of most other series with complete resolution approaching the 80% and satisfactory outcome exceeding the 85% with a low complication rate and no brainstem vascular insults despite the high complexity of the cases [2, 14, 15]. This elaborates on the effectiveness of the used decompression techniques as well as their safety ensured by the intraoperative ICG angiography without increasing the risk of associated complications. However, we should also stress that 15 patients are not a large number, and we recommend the adoption of this modality to our colleagues in order to have more patients to support its effectiveness.

Of course, it is not necessary to perform an ICG angiography in all cases of MVD as most of these procedures are straightforward, and the compressing vessels whether AICA or PICA can be easily repositioned using small pieces of shredded Teflon without the compromise of any perforators. However, we think that the ICG angiography technique is of great value whenever a transposition of a vessel with adjacent perforators is needed especially but not only restricted to cases with vertebrobasilar dolichoectasia. Whenever the surgeon is in doubt about the blood flow within a perforator and in cases of complex posterior fossa anatomy and dolichoectasia of the vertebrobasilar arterial system, ICG angiography should be used.

## Conclusions

Intraoperative indocyanine green angiography is a safe modality that might be helpful in avoiding brainstem ischemia while evaluating blood flow in small perforating arteries while performing microvascular decompression for hemifacial spasm. The great value of ICG angiography can be depicted in technically demanding MVD mostly for the decompression of vertebrobasilar dolichoectatic vessels using a sling technique.

## Supplementary Information

Below is the link to the electronic supplementary material.

Video 1 Showing live time intraoperative ICG angiography during MVD for a patient with dilochoectatic vertebral artery decompressed by VA transposition using the sling technique. The first part shows delayed filling of the perforator (arrowhead) after the initial application of the aneurysmal clip, and the second part shows timely filling of the perforator following readjustment of the clip and loosening of the sling and consequent less tension on the perforator
Supplementary file1 (MPG 62024 KB)
