# Correction to: The value of intraoperative indocyanine green angiography in microvascular decompression for hemifacial spasm to avoid brainstem ischemia

**DOI:** 10.1007/s00701-022-05427-z

**Published:** 2022-11-28

**Authors:** Ahmed Al Menabbawy, Ehab El Refaee, Loay Shoubash, Marc Matthes, Henry W. S. Schroeder

**Affiliations:** 1 Department of Neurosurgery, University Medicine, Greifswald, Greifswald, Germany; 2 Department of Neurosurgery, Cairo University, Giza, Egypt

**Correction to** **:** **Acta Neurochirurgica**

**https://doi.org/10.1007/s00701-022-05389-2**

The article "The value of intraoperative indocyanine green angiography in microvascular decompression for hemifacial spasm to avoid brainstem ischemia" written by Ahmed Al Menabbawy et. al, was originally published online October 25, 2022 has an error.

At the last sentences of paragraph 2 in the discussion; “Although most of the compressions are caused by arterial conflicting vessels (usually PICA or AICA), we had 2 cases of pure venous compressions, and some series reported up to 4.5% of cases with venous compression as the conflicting vessel “.

The mistake is that we had only one pure venous compression and not two. The second case with venous compression was actually combined with vertebral artery and was not pure. So, the correct statement should be; “Although most of the compressions are caused by arterial conflicting vessels (usually PICA or AICA), we had only one case of pure venous compression and another case with venous compression combined with vertebral artery compression, and some series reported up to 4.5% of cases with venous compression as the conflicting vessel.”

The original version of this article is corrected.

